# Supplementary material for: A set of multi-entry identification keys to African frugivorous flies (Diptera, Tephritidae)
Source: Zookeys. 2014 Jul 24;(428):97–108. doi: 10.3897/zookeys.428.7366 (PMC4143993; doi:10.3897/zookeys.428.7366)
Supplement: Supplementary material 6 — Key to Ceratitis [file zookeys-428-097-s006.zip › SF6_ZooKeys_key to Ceratitis/key/SF6_ZooKeys_key to Ceratitis/Media/Html/Ceratitis discussa .htm]

Ceratitis (Ceratalaspis) discussa Munro


***Ceratitis*** **(*Ceratalaspis*)
*discussa* Munro**

*Ceratitis* (*Pardalaspis*) *discussa*
Munro, 1935: 307.

Body
length: 5.42 (5.00-5.85) mm; wing length: 4.93 (4.40-5.40) mm.

Male

Head.
Antenna yellow-orange. Third antennal segment twice as long as second segment.
Arista with short hairs over entire length. Frons with short scattered hairs
which are same colour as frons; more flattened, not distinctly convex, in
lateral view slightly projecting at antennal implant. Lower eye margin at most
with slightly darker yellow marking. Chaetotaxy normal for subgenus, dark
reddish.

Thorax.
Postpronotum white, with black spot. Ground colour of mesonotum pale with
yellow tinge; mesonotum: spots at mesal end of transverse suture sometimes
smaller and/or only darkish brown, anterior supra-alar spots usually at least
partly interrupted. Chaetotaxy normal for subgenus. Scapular setae pale. One
anepisternal bristle. Scutellum white basally, otherwise yellow with three
black separate markings apically; basally without dark spots, at most darker
yellow colouration. Subscutellum pale with three dark separate spots, sometimes
touching along dorsal margin.

Legs
yellow; setation typical for subgenus, mainly pale especially on femora.
Posterior and posterodorsal rows on fore femur pale. Ventral spines on fore
femur pale.

Wing
bands with markings extensively yellow. Banding, marginal band continuous;
cubital band free; medial band absent; crossvein r-m before middle of discal
cell. Crossvein dm-cu posteriorly more inwards than anteriorly.

Abdomen.
Pale yellow. Setation and banding typical for subgenus.

Female

As
male except for the following characters: Third antennal segment somewhat
longer, almost three times as long as second segment. Frons with scattered
hairs which are darker than frons. Crossvein r-m sometimes at middle of discal
cell. Oviscape shorter than abdominal terga 3-6 combined.

(Description
after De Meyer, 1998)
